# MicroRNA-936 inhibits the malignant phenotype of retinoblastoma by directly targeting HDAC9 and deactivating the PI3K/AKT pathway

**DOI:** 10.3892/or.2022.8389

**Published:** 2022-08-18

**Authors:** Lishuai Xu, Weidong Li, Qian Shi, Minfeng Wang, Heng Li, Xiaoli Yang, Junjun Zhang

Oncol Rep 43: 635–645, 2020; DOI: 10.3892/or.2020.7456

Following the publication of the above paper, it was drawn to the Editors' attention by a concerned reader that certain of the flow cytometric data featured in Figs. 2D, 4D and 5D, the colony formation assay data shown in Figs. 2C, 4C and 5C, and the tumor images shown in Fig. 7A were strikingly similar to data appearing in different form in other articles by different authors. Owing to the fact that the contentious data in the above article were already under consideration for publication, or had already been published, prior to its submission to *Oncology Reports*, the Editor has decided that this paper should be retracted from the Journal. The authors were asked for an explanation to account for these concerns, but the Editorial Office did not receive a reply. The Editor apologizes to the readership for any inconvenience caused.

